# Prevalence of Asymptomatic SARS-CoV-2 Infection in the General Population of the Veneto Region: Results of a Screening Campaign with Third-Generation Rapid Antigen Tests in the Pre-Vaccine Era

**DOI:** 10.3390/ijerph182010838

**Published:** 2021-10-15

**Authors:** Silvia Cocchio, Michele Nicoletti, Francesco Paolo De Siena, Gaia Lattavo, Patrizia Furlan, Marco Fonzo, Michele Tonon, Federico Zabeo, Francesca Russo, Vincenzo Baldo

**Affiliations:** 1 Department of Cardiac Thoracic and Vascular Sciences, and Public Health, University of Padua, 35131 Padua, Italy; silvia.cocchio@unipd.it (S.C.); michele.nicoletti@studenti.unipd.it (M.N.); francescopaolo.desiena@studenti.unipd.it (F.P.D.S.); gaia.lattavo@studenti.unipd.it (G.L.); patrizia.furlan@unipd.it (P.F.); marco.fonzo@unipd.it (M.F.); federico.zabeo@unipd.it (F.Z.); 2 Regional Directorate of Prevention, Food Safety, Veterinary Public Health—Regione del Veneto, 30123 Venice, Italy; michele.tonon@regione.veneto.it (M.T.); francesca.russo@regione.veneto.it (F.R.)

**Keywords:** SARS-CoV-2, COVID-19, surveillance, public health, epidemiology

## Abstract

The aim of our study was to ascertain the prevalence of SARS-CoV-2 infection in the general population during a period of moderate risk, just before Italy started to implement its vaccination campaign. A third-generation antigenic nasal swab sample was collected by a healthcare provider, and all individuals testing positive subsequently had a nasopharyngeal swab for molecular testing; the result was used to calculate the positive predictive value. The population consisted of 4467 asymptomatic adults with a mean age of 46.8 ± 16.00 years. The 62.2% tested for the first time, while 37.8% had previously undergone a mean 2.2 tests for SARS-CoV-2. With 77 of our overall sample reporting they had previously tested positive for COVID-19 and 14 found positive on our screening test, the overall estimated prevalence of the infection was 0.31%. Nine of the 14 cases were confirmed on molecular testing with a PPV of 64.3%. The mean age of the individuals testing positive was 38.1 ± 17.4. Based on the timing of symptom onset, six of the above cases were classified as false negatives, and the adjusted estimated prevalence was 0.34%. Describing levels of infection in a general population seems to be very difficult to achieve, and the universal screening proved hugely expensive particularly in a low-prevalence situation. Anyway, it is only thanks to mass screening efforts that epidemiological data have been collected. This would support the idea that routine screening may have an impact on mitigating the spread of the virus in higher-risk environments, where people come into contact more frequently, as in the workplace.

## 1. Introduction

On 31 December 2019, China reported a pneumonia cluster caused by an infection of unknown etiology [1]. Over subsequent weeks, the pathogen was named SARS-CoV-2, and the syndrome it caused COVID-19. On 30 January 2020, the World Health Organization (WHO) classified this infection as a Public Health Emergency of International Concern [2].

Italy declared a state of emergency on 1 February [3], and the government signed the first containment strategies soon afterwards. The Italian experience of COVID-19 can be divided into three phases. The first, from the beginning of March to the end of May 2020, coincided with a high transmission rate in the north of the country. Then, the number of new infections remained low from July to early September 2020. Starting in the autumn, the epidemiological pattern evolved completely, and there was a huge surge in the infection rates [4] that put the national health system under very severe strain (too many citizens needed oxygen therapy, and critical care beds were nearly all occupied). The Prime Minister introduced various limitations on individual freedom of movement [5], and the infection rates gradually fell almost everywhere in Italy [4]. Travelling between regions was forbidden from 21 December to 6 January 2021, and citizens were obliged to stay within their home towns for Christmas and New Year [6].

More than one in two of all cases of infection with SARS-CoV-2 to date were recorded in four regions of northern Italy: Lombardy, Piedmont, Veneto and Emilia Romagna [7]. Such a localized pattern in the diffusion of the virus prompted national policies that divided Italy into “areas” with the aim of stratifying the emergency response and, thus, sustaining the economy in regions where hospitals were not under strain [8]. This so-called “traffic light” approach involved classifying the areas most in need (from a public health standpoint) as red and those coping better as yellow or green. The Veneto region was at “medium-risk” [9], with an estimated reproduction number (Rt) of 0.95, and was initially classified as “orange”. From the start of 2021 until early March, citizens were allowed to move within their own home towns from 5.00 to 22.00 h, and any travelling across municipal boundaries was only permitted for essential workers and health-related reasons. Schools were open, but with only half the students attending at the same time.

The number of infections recorded during Italy’s second wave of COVID-19 infection (in late 2020 and early 2021) was much greater than during the first in spring 2020 [4]. This was probably due largely to the huge number of swabs performed on asymptomatic subjects (who were not tested during the first peak). The introduction of the antigen test also enabled far more individuals to be screened. The testing strategy was based on a first-level swab for antigen testing so that molecular tests could be used for symptomatic citizens or individuals found positive to the antigen test. In short, very large numbers of individuals had antigen tests, the majority not prompted by clinical issues, but as a form of screening.

The aim of our study was to ascertain the prevalence of SARS-CoV-2 infection in the general population during a period of moderate risk, just before Italy started to implement its vaccination campaign. In January 2021, we tested only people with no symptoms to obtain a detailed picture of the infection risk in everyday life for a population wearing masks and complying with social and physical distancing rules.

## 2. Materials and Methods

### 2.1. Study Population

Study participants were enrolled from the general population of the Veneto region between 8 and 28 January 2021. The test settings were chosen to ensure a high flow of people and maximize the numbers that could be tested within a reasonably short time. Participants were drawn from among workers and customers at supermarkets and shopping centers, Italian Red Cross (IRC) voluntary workers and employees of local authorities and the Italian Economy and Finance Ministry (MMEF). To be included in the study, they had to be at least 18 years old and have experienced no plausibly COVID-19-related symptoms in the previous 5 days. Participants were tested only once, after signing an informed consent form.

All participants provided socio-demographic data, including the province where they lived, their occupation and their education level. They were asked about any clinical issues and comorbidities, any onset of possibly COVID-19-related symptoms and whether and why they had already been tested for COVID-19. They also provided information about their routine behavior and lifestyle to estimate the number of their daily contacts and how many times a day they usually went out.

The study population was, then, divided into two subgroups: one consisted of individuals who had been previously screened with at least one nasal/nasopharyngeal test; the other included those never previously tested.

### 2.2. Testing Procedure

A nasal swab sample was collected by a healthcare provider (HCP) from both nasal cavities. The test to identify SARS-CoV-2 was performed using a microfluidic immunofluorescence assay for the qualitative detection of nucleocapsid antigens to SARS-CoV-2 (Lumira DX). This type of test requires a disposable test strip and a reader. The sample collected is placed in an extraction tube where a single drop of extracted sample is applied to the strip. Results are available after approximately 12 min. At the pre-marketing stage, manufacturers declared a sensitivity of 97.6% and a specificity of 96.6%, but a recent meta-analysis on the clinical accuracy of novel rapid antigen diagnostics for SARS-CoV-2 set the pooled sensitivity and specificity at 88.2% (59.0–97.5) and 98.6% (96.2–99.5), respectively [10].

All individuals testing positive subsequently had a nasopharyngeal swab for molecular testing, and the result was used to calculate the positive predictive value (PPV) of the antigen test.

### 2.3. Data Analysis

All tests were recorded in a database managed by the Veneto Regional Authority and individuals testing positive were reported to the local services managing the appropriate preventive measures. A unique anonymized ID code was generated for each participant recorded in the database, which enabled them to be followed up for the purposes of the study. All tests they underwent in the 14 days after their enrollment were available. For individuals found negative at our screening but positive on a molecular test performed during the two weeks thereafter, we collected information on the reason for the molecular test, when it was performed and the clinical picture at the time.

### 2.4. Statistical Analysis

The data were analyzed using the chi-square test as appropriate estimating the ORs and the confidence interval 95% (CI95%). Age was summarized as medians and interquartile ranges (IQR), and comparisons were made using the Wilcoxon rank sum test. Statistical analyses were performed using the SPSS version 27.0. A *p*-value less than 0.05 was considered statistically significant. The sample size was obtained using a prior prevalence for SARS-CoV-2 of 0.4%, a marginal error of 0.2% and a type 1 error of 5%—two sided: a minimum sample of 3800 people was needed (EpiInfo Software).

## 3. Results

The population included in the study consisted of 4467 asymptomatic adults: 62.2% (2780 subjects) tested for the first time, while 37.8% (1687 subjects) had previously undergone a mean 2.2 tests for SARS-CoV-2 (median: 1, IQR 25%: 1 and IQR 75%: 2), for a total of 3650 tests, 58.1% molecular. Table 1 shows the characteristics of the study population for the two groups. There were no statistically significant differences in gender distribution (48.9% males) between the two. The mean age of the sample as a whole was 46.8 ± 16.00 years (46.5 ± 15.9 for males and 47.2 ± 16.2 for females). The previously screened group was slightly younger, with a mean age of 44.9 ± 15.5 years, (median: 48, IQR 25%: 34 and IQR 75%: 59) as opposed to 48.0 ± 16.3 years for the group screened for the first time (median: 49, IQR 25%: 34 and IQR 75%: 57) (*p* < 0.001).

In the previously screened group, 61.2% of the subjects claimed to meet more than five people a day for work-related reasons, while this was true of 56.2% of those screened for the first time [*p* = 0.001, OR = 1.23 (CI 95%: 1.09–1.39)]. Much the same pattern emerged for how many times a day individuals usually left home: 75.0% of participants in the previously screened group said they routinely went out at least once a day, as opposed to 70.3% of the group not previously screened [*p* = 0.001, OR = 1.27 (CI 95%: 1.11–1.46)].

With 77 (1.7%) of our overall sample reporting they had previously tested positive for COVID-19, and 14 found positive on our screening test, the overall estimated prevalence of the infection was 0.31%. Nine of the 14 cases were confirmed on molecular testing, so the corrected prevalence dropped to 0.20% CI 95%(0.07–0.33) (9/4467), and the PPV was 64.3% CI 95%(39.2–89.4). Table 2 summarizes the characteristics of the 14 subjects tested positive and grouped by the result of molecular testing.

The corrected prevalence of the infection among the individuals screened for the first time was estimated at 0.22% (6/2780), while for the previously screened group, it was 0.18% (3/1687) (*p* is not statistically significant). The mean age of the individuals testing positive was 38.1 ± 17.4, with no statistically significant differences by gender. Two of the individuals testing positive worked in a supermarket or shopping center, one volunteered with the Italian Red Cross, one was employed by a local authority, and one was a student; no job-related information was available for the other four.

After being notified they had tested positive, five individuals reported feeling ill: three with headache, muscle pain and loss of taste; two others had fever and loss of smell; one complained of sore throat; one reported having already experienced some symptoms in the previous week.

**Figure 1:**
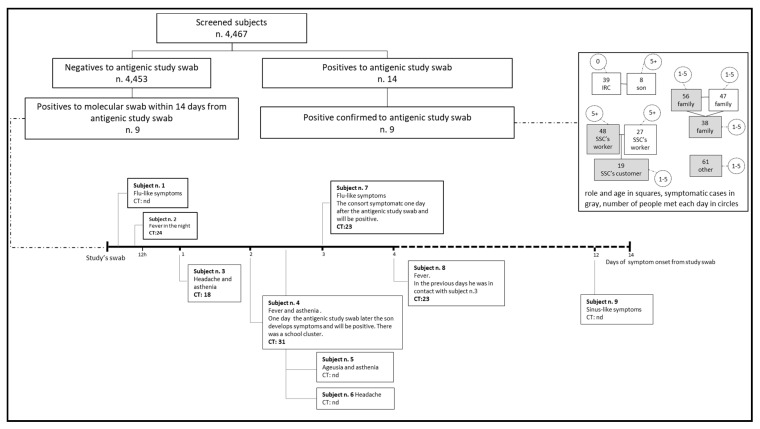
Analysis of individuals found positive, their symptom onset timelines and clustering of cases. Abbreviations: CT—cycle threshold; IRC—Italian Red Cross; SSC—supermarkets and shopping centers.

The work carried out by the contact tracing units enabled us to establish that eight of the nine infected individuals belonged to three different clusters, two of which developed in family settings and one in a workplace (Figure 1).

In the two weeks after our screening, nine participants who tested negative with the antigen test were found positive on molecular testing. Figure 1 shows the date of symptom onset (if reported at the time of our screening test) and the cycle threshold (CT) of their molecular test, where available. Two of these nine participants started experiencing symptoms some hours after our antigen test—one in the afternoon, the other during the night (with a CT of 24). One participant reported headache and fatigue the next day (CT 18); it is worth mentioning that this individual worked with another participant, who started to feel ill 4 days after our test. Another two participants (with a CT of 31 and 23) developed symptoms and were found positive soon after a housemate had tested positive. Based on the timing of symptom onset vis-à-vis our antigen test, six of the above cases were classified as false negatives. Adding these six cases to the nine found positive by our antigen test and confirmed by a subsequent molecular test, the adjusted estimated prevalence was 0.34% (15/4.467).

## 4. Discussion

Our cross-sectional study offers a snapshot of SARS-CoV-2 infection in the general population in Italy in January 2021, when the measured point prevalence (the proportion of people with a disease or condition at a given time) stood at 0.20%. Although comparisons with other experiences must be drawn with caution due to the undeniable influence of different settings, restrictions implemented, test methods used and the phase of the epidemic in a given area, our finding falls within the range of figures obtained in other, similar studies conducted on representative samples of general resident populations. The combined results of the first six phases of the English Real-time Assessment of Community Transmission (REACT) program found a 0.3% prevalence of SARS-CoV-2 positivity [11], and so did a survey of English residents conducted by Petersen and Phillips between 26 April and 27 June 2020 [12]. On the other hand, our result is significantly lower than that of a cross-sectional study conducted in Iceland in March–April 2020, where the prevalence was 0.6–0.8% [13].

Public health practitioners have focused on strategies to identify the best COVID-19 testing strategies. Until now, the debate has revolved around whether to use antigen tests, or how to establish their efficacy. Few analyses have examined the real rate of infection in the population. In the absence of a known epidemiological link, the assumption of SARS-CoV-2 negativity has always relied on the lack of symptoms. Intriguingly, this assumption has been used explicitly in studies designed to compare COVID-19 transmission patterns between symptomatic and asymptomatic individuals [14].

Preliminary findings of economic models suggest that screening tests and social distancing are effective in preventing and controlling COVID-19 transmission on a long-term horizon, but universal screening proved hugely expensive. Cost-effectiveness studies have generally indicated that such efforts could only be justified by monetizing the gain in QALY [15,16]. Currently available evidence still seems insufficient and too heterogeneous to support any definitive conclusions regarding the costs of interventions, and further research in this direction should be encouraged.

We could find no data or studies on the accuracy of the outcomes of serial screening strategies. The prevalence of COVID-19 infection among the low-risk general population proved extremely low, confirming data obtained by mass testing campaigns. This poses a problem inasmuch as a low prevalence means that the sensitivity of a screening test must be high to achieve acceptable predictive values [14]. In our study, using a point-of-care tool seemed to have more appeal for the general public and made it easier to test a reasonable number of people at every session. The use of nasal swabs and a semi-automatic laboratory machine reduced the risk of operator-dependent errors and kit failures.

### Strengths and Limitations

Describing levels of infection in a general population seems to be very difficult to achieve, and this is also true of our study. People who have felt ill or whose behavior has exposed them to greater risk are more likely to come forward for screening because of their perception of a moderate-to-high personal risk. On the other hand, it is quite hard to test low-risk population groups, and it is only thanks to mass screening efforts that statistical data have been collected [12,13,17]. As in the case of any other infectious disease, some infected individuals go undetected, and the level of transmission may be underestimated [18]. On the other hand, our study is one of the few to have estimated the prevalence of SARS-CoV-2 in a substantial sample of the general population (enrolling more than 4000 individuals). It is also one of the last to produce evidence of the diffusion of SARS-CoV-2 before vaccination campaigns started around the world.

As previously mentioned in the Results section, no statistically significant difference emerged in the prevalence of SARS-CoV-2 positivity between previously screened individuals and those coming forward for testing for the first time. This is despite the fact that the two groups differed substantially in terms of potential exposure to the virus: the previously screened individuals were 23% more likely to have five or more contacts a day and 27% more likely to leave the house at least once a day, compared with those screened for the first time. This difference probably had to do with work-related needs, given the restrictions on people’s movements in place at the time of our study.

## 5. Conclusions

The routine screening—along with pharmacological and other interventions—may have an impact on mitigating the spread of the virus in higher-risk environments, where people come into contact more frequently, as in the workplace. Although vaccine coverage is expanding both nationally and internationally, further research along the lines of the present study could be helpful in orienting prevention strategies in the event of any spread of vaccine-resistant variants.

## Figures and Tables

**Table 1:** Characteristics of the study population.

Characteristics	Study Population					
	Previously Screened (n. 1687)		Screened for First Time (n. 2780)		Total (n. 4467)	
	n	(%)	n	(%)		
Gender						
Male	853	(50.6)	1332	(47.9)	2185	(48.9)
Female	834	(49.4)	1448	(52.1)	2282	(51.1)
Age, median (IQR)	48 (34; 59)		49 (34; 57)		48 (34; 58)	
Age group						
<30	325	(19.3)	469	(16.9)	794	(17.8)
30–49	657	(38.9)	905	(32.6)	1562	(35.0)
50–69	621	(36.8)	1164	(41.9)	1785	(40.0)
70+	84	(5.0)	242	(8.7)	326	(7.3)
No. of people they met each day						
No one	146	(8.7)	326	(11.7)	472	(10.6)
1–5	508	(30.1)	891	(32.1)	1399	(31.3)
5+	1033	(61.2)	1563	(56.2)	2596	(58.1)
No. of times they went out each week						
≥1/day	1266	(75.0)	1953	(70.3)	3219	(72.1)
2–6/week	253	(15.0)	464	(16.7)	717	(16.1)
0–1/week	168	(10.0)	363	(13.1)	531	(11.9)

**Table 2:** Characteristics of the subjects tested positive for COVID-19 on screening test.

Characteristics	Subjects Tested Positive	
	Confirmed on Molecular Testing (n.9)	Not Confirmed on Molecular Testing (n.5)
Age (mean ± standard deviation)	38.1 ± 17.4	52.0 ± 6.4
Age group		
<30	3	
30–49	4	2
50–69	2	3
Gender		
Male	6	2
Female	3	3
Profession		
Workers at supermarkets and shopping centers	2	
Customers at supermarkets and shopping centers	5	3
Employees of local authorities	1	2
Italian Red Cross voluntary workers	1	

## Data Availability

The data supporting the findings of this study are available from the corresponding author upon reasonable request.
